# Enzymatic Biotransformation of Ginsenoside Rb_2_ into Rd by Recombinant α-L-Arabinopyranosidase from *Blastococcus saxobsidens*

**DOI:** 10.4014/jmb.1910.10065

**Published:** 2019-12-24

**Authors:** Ju-Hyeon Kim, Jung-Mi Oh, Sungkun Chun, Hye Yoon Park, Wan Taek Im

**Affiliations:** 1 Department of Biotechnology, Hankyong National University, Anseong 7579, Republic of Korea; 2 HK Ginseng Research Center, Hankyong National University, Anseong 17579, Republic of Korea; 3 Department of Physiology, Chonbuk National University Medical School, Jeonju 54907, Korea; 4 National Institute of Biological Resources, Incheon 22689, Republic of Korea; 5 AceEMzyme Co., Ltd., Anseong 1779, Republic of Korea

**Keywords:** Ginsenoside Rb_2_, ginsenoside Rd, biotransformation, α-L-arabinopyranosidase, *Blastococcus saxobsidens*

## Abstract

In this study, we used a novel α-L-arabinopyranosidase (AbpBs) obtained from ginsenosideconverting *Blastococcus saxobsidens* that was cloned and expressed in *Escherichia coli* BL21 (DE3), and then applied it in the biotransformation of ginsenoside Rb_2_ into Rd. The gene, termed *AbpBs*, consisting of 2,406 nucleotides (801 amino acid residues), and with a predicted translated protein molecular mass of 86.4 kDa, was cloned into a pGEX4T-1 vector. A BLAST search using the AbpBs amino acid sequence revealed significant homology with a family 2 glycoside hydrolase (GH2). The over-expressed recombinant AbpBs in *Escherichia coli* BL21 (DE3) catalyzed the hydrolysis of the arabinopyranose moiety attached to the C-20 position of ginsenoside Rb_2_ under optimal conditions (pH 7.0 and 40°C). Kinetic parameters for α-Larabinopyranosidase showed apparent K_m_ and V_max_ values of 0.078 ± 0.0002 μM and 1.4 ± 0.1 μmol/min/mg of protein against *p*-nitrophenyl-α-L-arabinopyranoside. Using a purified AbpBs (1 μg/ml), 0.1% of ginsenoside Rb_2_ was completely converted to ginsenoside Rd within 1 h. The recombinant AbpBs could be useful for high-yield, rapid, and low-cost preparation of ginsenoside Rd from Rb_2_.

## Introduction

Ginseng has been used as a traditional herbal medicine to cure diseases and promote health in Asian countries for thousands of years, but has also gained recognition in the past decade in the West for its beneficial uses [1-3]. Many previous reports have shown that ginseng has extensive pharmacological and therapeutic effects on humans including anticancer [4], anti-inflammatory activity [5], neuro-protective effects [6], anti-amnestic [7], immuno-modulatory [8], and radio protective properties [9]. Most medicinal effects of ginseng have been attributed to triterpene saponins, also referred to as ginsenosides. [10, 11]. Hence, more than 180 kinds of naturally occurring saponins have been isolated for pharmaceutical usage [12].

Ginsenoside Rd, a major ginsenoside in ginseng, has shown inhibitory effect on carrageenan-induced inflammation [13], promotive effect on neural stem cells [14], and wound-healing effect [15]. Ginsenoside Rd is structurally similar to Rb_1_, Rb_2_, and Rc but lacks one outer glycoside moiety at position C20. Therefore, Rb_1_, Rb_2_, and Rc can be transformed into Rd by cleavage of the outer glucose, arabinopyranose or arabinofuranose moieties by β-glucosidase, α-L-arabinofuranosidase, and α-L-arabinopyranosidase, respecti-vely [16, 17].

**Fig. 1:**
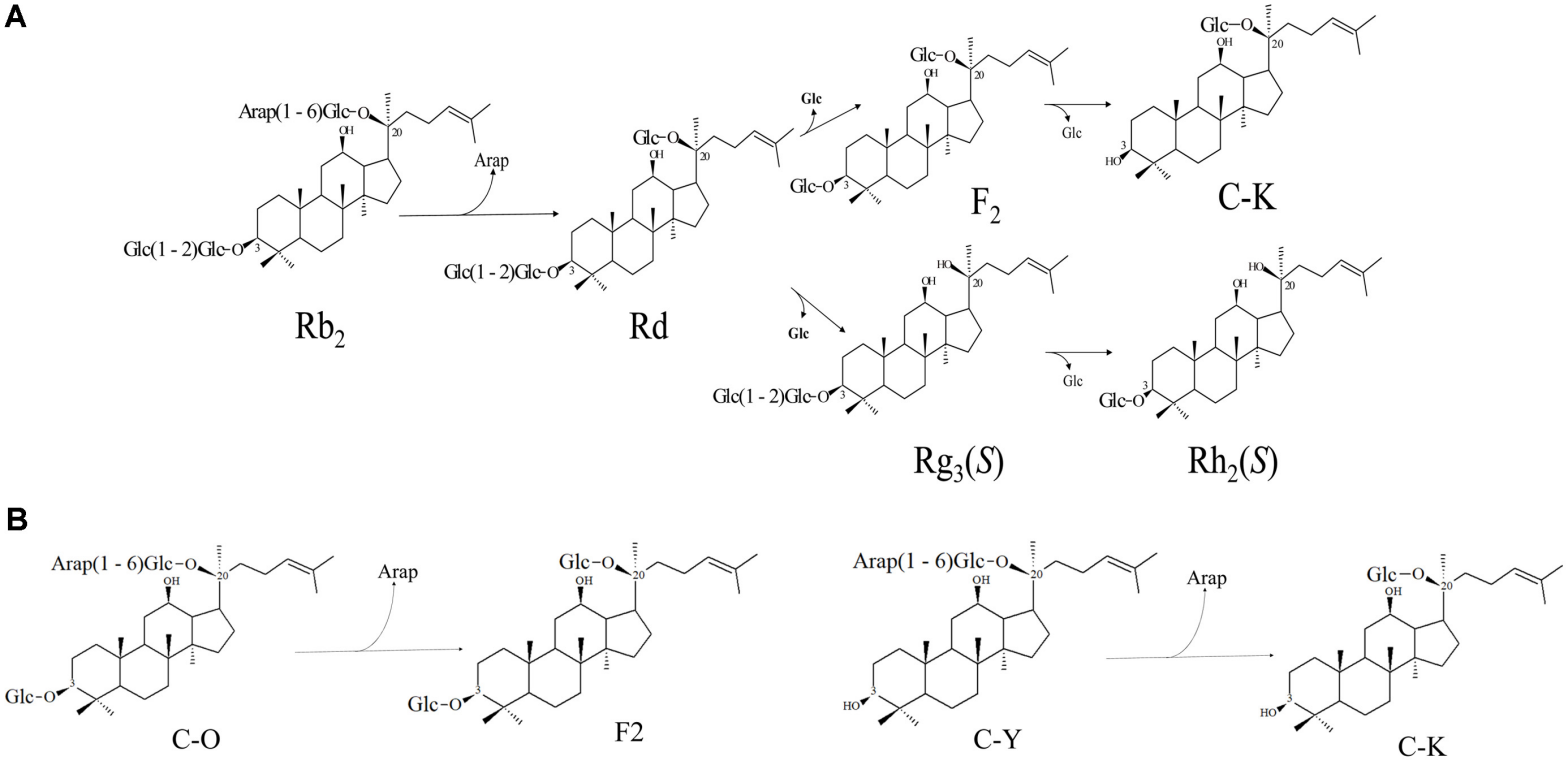
(**A**) Biotransformation pathway of ginsenoside Rb_2_ into Rd, which is the important divergent position of the biotransformation of ginsenoside; (**B**) biotransformation pathway of ginsenoside C-O, C-Y into F_2_, C-K, respectively, by α-Larabinopyranosidase (AbpBs).

Also among the major ginsenosides, ginsenoside Rb_2_ comprises 1-22% of the total ginsenosides in ginseng root [18, 19], and is thus required for conversion into Rd, which is an important divergent position in the biotransformation of ginsenoside: Rd → Rg_3_(*S*) → Rh_2_(*S*) pathway or Rd → F_2_ → compound K pathway (Fig. 1A). One solution to this then is to obtain recombinant α-L-arabinopyranosidase to biotransform ginsenoside Rb_2_ into Rd with high activity (Fig. 1). Up to now, several methods have been developed for producing minor ginsenosides such as heating, acid treatment and enzymatic methods [17,20-22]. The enzymatic methods are considered as the most promising approach, the advantages being fewer byproducts, better environmental protection, and better stereo-specificity [23].

In this study, we report the cloning and characterization of a novel ginsenoside-transforming α-L-arabinopyranosidase (AbpBs) from *Blastococcus saxobsidens*, followed by expression in *Escherichia coli* and characterization of α-Larabinopyranosidase (AbpBs). AbpBs belongs to glycoside hydrolase family 2, and this recombinant enzyme could efficiently catalyze the conversion of ginsenoside Rb_2_ to Rd by selectively hydrolyzing the outer arabinopyranoside moiety at the C20 position. In the same way, this enzyme could also hydrolyze ginsenoside compound O (C-O) and compound Y (C-Y) into ginsenoside F_2_ and compound K (C-K), respectively (Fig. 1B).

## Materials and Methods

### Materials

Ginsenosides Rb_1_, Rb_2_, Rc, Rd, Re, Rg_3_(*S*), F_1_, F_2_, protopanaxadiol and C-K were purchased from AceEMzyme Co., Ltd. (Korea). Chromogenic substrates for an enzyme activity assay were obtained from Sigma. *Blastococcus saxobsidens* KACC 20608^T^ used for cloning α-L-arabinopyranosidase gene was cultivated on LB agar (BD, USA), under aerobic condition at 30°C. *Escherichia coli* BL21 (DE3) and pGEX-4T-1 plasmid (GE Healthcare, USA), for gene cloning and expression, were cultivated in a Luria–Bertani (LB) medium supplemented with ampicillin (70 mg/l). The other chemicals used in this study were at least of analytical reagent grade, and the sources are noted individually in the methods section below.

### Analysis of AbpBs Sequence

Database homology search was performed with the BLAST program provided by NCBI. Furthermore, the multiple amino acid sequence alignment and the conserved patterns of discrete amino acid sequences of AbpBs and the known, most homologous α-L-arabinopyranosidase were performed by using the ClustalW program. (http://embnet.vital-it.ch/software/ClustalW.html).

### Cloning, Expression, and Purification of Recombinant AbpBs

Genomic DNA of *Blastococcus saxobsidens* KACC 20608^T^ was extracted using a genomic DNA extraction kit (Macrogen, Korea). The gene, termed *AbpBs*, and encoding α-L-arabinopyranosidase,(GenBank Accession No. WP_104529000.1) was amplified by polymerase chain reaction (PCR) using Pfu DNA polymerase (BIOFACT, Korea) and the following primers containing BamHI and XhoI restriction sites (underlined): AbpBsF (5’- GGT TCC GCG TGG ATC CAT GCG ACG CAT CCC CTT C-3’) and AbpBsR (5’- GAT GCG GCC GCT CGA GTC ATC GAG CCT CGA TTC C-3’). The amplified DNA fragments were purified and inserted into a pGEX 4T-1 glutathione S-transferase (GST) fusion vector using an EzCloning Kit (Enzynomics Co. Ltd., Korea) to generate a GST-*AbpBs* fusion gene. *E. coli* BL21 (DE3), transformed with recombinant pGEX-AbpBs, was grown in LB-ampicillin medium at 37°C until the culture reached an OD_600_ of 0.5, at which point protein expression was induced by adding 0.1 mM isopropyl-β-D-thiogalactopyranoside (IPTG). Additionally, bacteria were incubated for 18 h at 20°C with shaking at 200 rpm and then harvested by centrifuging at 10,000 ×*g* for 20 min at 4°C. The cells were washed twice with 50 mM sodium phosphate buffer (pH 7.0, 5 mM EDTA, and 1% Triton X-100) and then suspended in 50 mM sodium phosphate buffer (pH 7.0). In order to obtain crude cells, the cells were disrupted by ultrasonication (Vibra-Cell, USA) on ice at 5 min and the intact cells and debris were removed by centrifugation at 13,000 ×*g* for 20 min at 4°C. The GST-tagged fusion protein was purified by GST-bind agarose resin (Elpis, Korea). The GST tag was removed from the resin after incubation with thrombin. The homogeneity of the protein was assessed by 10% sodium dodecyl sulfate polyacrylamide gel electrophoresis (SDS-PAGE) and EzStain AQUA (Atto, Japan).

### Enzyme Characterization and Determination of Kinetic Parameters

To determine the optimum conditions for activity, pH, temperature, metal ions and chemical reagents were investigated as previously described [24]. The substrate specificity of AbpBs was tested using 2 mM *p*-nitrophenyl (*p*NP)- and *o*-nitrophenyl (*o*NP)-glycosides with α and β configurations described by [25]. The specific activity of purified AbpBs was determined using *p*-nitrophenyl-α-L-arabinopyranoside (*p*NP-α-L-arabinopyranoside) as substrate in 50 mM sodium phosphate buffer (pH 7.0) at 40°C. *p*-Nitrophenol release was immediately measured using a microplate reader at 405 nm (Bio-Rad Model 680, USA). One unit of activity was defined as the amount of protein required to generate 1 μmol of p-nitrophenol per minute. Protein concentrations were determined using the bicinchoninic acid (BCA) protein assay (Pierce, USA), with bovine serum albumin (Sigma) as the standard. All assays were performed in triplicate. Kinetic studies were performed using freshly purified enzyme (10 μg/ml), and *p*NP-α-L-arabinopyranoside and ginsenoside Rb_2_ at concentrations ranging from 0.1 mM to 5 mM. All enzyme assays were performed in triplicates, and the parameters were determined as described by Cleland [26].

### Enzymatic Hydrolysis of Ginsenosides

To investigate the biotransformation ability of recombinant AbpBs, three kinds of ginsenosides (Rb_2_, compound O, compound Y) having outer arabinopyranose moiety at C20 were evaluated as substrates. Initial biotransformation experiments using ginsenoside Rb_2_ as substrate revealed that GST fused with AbpBs did not affect the activities of AbpBs. Each assay unit was composed of ginsenoside and fused protein solution (0.2 mg/ml in 50 mM sodium phosphate buffer, pH 7.0) in a 1:1 ratio (v/v) at 37°C. In addition, the hydrolyzing capacity of AbpBs (10 μg/ml) was determined using 2.0 mg/ml of Rb_2_, compound O and compound Y, respectively, as substrates in 50 mM sodium phosphate buffer (pH 7.0) at 40°C. Samples were withdrawn at regular intervals and an equal volume of water-saturated *n*-butanol was added to stop the reaction, and the reactant present in the *n*-butanol fraction was analyzed by TLC after pretreatment.

### Analytical Methods

**TLC analysis.** A reaction solution containing ginsenoside was extracted with an equal volume of water-saturated *n*-butanol; after centrifugation, the *n*-butanol fraction was examined by TLC using 60F_254_ silica gel plates (Merck, Germany) and CHCl_3_-CH_3_OH-H_2_O (65:35:10, v/v/v, lower phase) as the solvent. TLC plates were sprayed with 10% (v/v) H_2_SO_4_, followed by heating at 110°C for 5 min to visualize ginsenoside spots, which were identified by comparing with a standard.

**HPLC analysis.** HPLC analysis of ginsenosides was performed using AutoChro 3000 software (Younglin, Korea) equipped with a quaternary pump, automatic injector, and single-wavelength UV detector (model 730D) for peak identification and integration. The HPLC column used was a Prodigy ODS (2) C18 column (4.6 × 150 mm, 5 μm) (Phenomenex, USA) combined with an Agilent safeguard column. Isocratic elution was performed, using acetonitrile (**A**) and water (**B**) at a ratio of 34:66 (v/v) as mobile phase, for 20 min, at a flow rate of 1.0 ml/min. Detection was performed by monitoring absorbance at 203 nm.

## Results and Discussion

### Analysis of AbpBs Sequence

The α-L-arabinopyranosidase gene (*AbpBs*) consists of 2,406 bp encoding 801 amino acids with a molecular mass of 86.4 kDa and a theoretical pI value of 5.00 (http://web.expasy.org/compute_pi/). AbpBs has homology to the protein domain of glycoside hydrolase family 2 (GH2) of which enzyme activity showed β-galactosidase (E.C. 3.2.1.23), β-mannosidase (E.C. 3.2.1.25), β-glucuronidase (E.C. 3.2.1.31), α-L-arabinopyranosidase (E.C. 3.2.1.-), and β-xylosidase (E.C. 3.2.1.37). The Carbohydrate-Active enZymes database (http://www.cazy.org) describes more than 17,851 uncharacterized and 185 characterized GH2 members that are widespread across numerous organisms. However, just one α-L-arabinopyranosidase gene was annotated from *Bacteroides thetaiotaomicron* VPI-5482 in characterized GH2 members. To the best of our knowledge, AbpBs had not been characterized yet before this research. AbpBs is homologous to the α-L-arabinopyranosidase in *Geodermatophilaceae* bacterium URHB0048 (GenBank Accession No. WP_029336779, 83.3%), *Modestobacter marinus* (WP_014740221, 74.7%), and *Streptomyces fulvoviolaceus* NRRL B-2870 (WP_030618049, 60.1%) based on its amino acid sequence similarities.

### Cloning, Expression, and Purification of Recombinant AbpBs

**Fig. 2:**
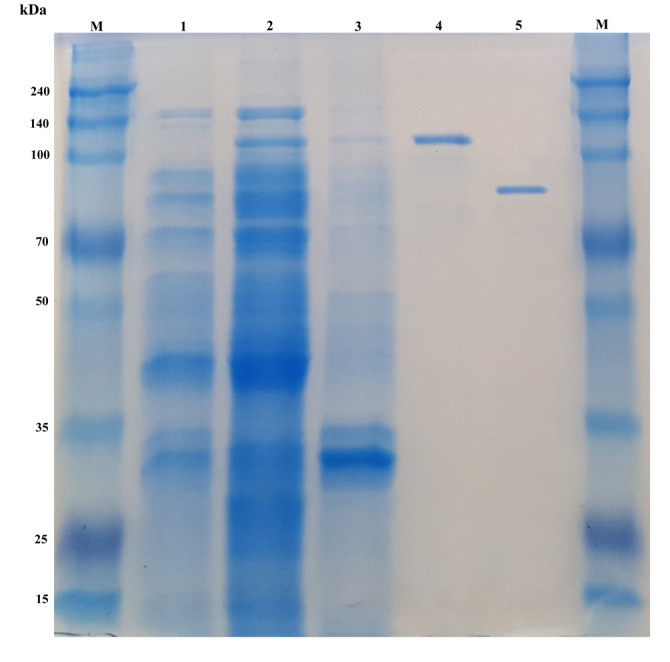
Purification of recombinant AbpBs: M, molecular mass markers; lane 1, crude extract of uninduced *E.coli* BL21(DE3); lane 2, soluble crude extract; lane 3, precipitated crude extract lane 4, fusion protein showing GST-AbpBs; lane 5, purified AbpBs after treatment with thrombin.

GST-AbpBs was expressed in *E. coli* BL21 (DE3). To maximize the yield of the fusion protein in soluble form, we tested different induction conditions and found that induction with 0.1 mM IPTG at 20°C for 24 h produced a half-soluble active fusion enzyme. Supernatant from the cell lysates and purified protein samples were applied to SDS–PAGE. The calculated molecular mass of the AbpBs (86.4 kDa) was similar to the mass detected via SDS-PAGE (Fig. 2).

### Enzyme Characterization

**Fig. 3:**
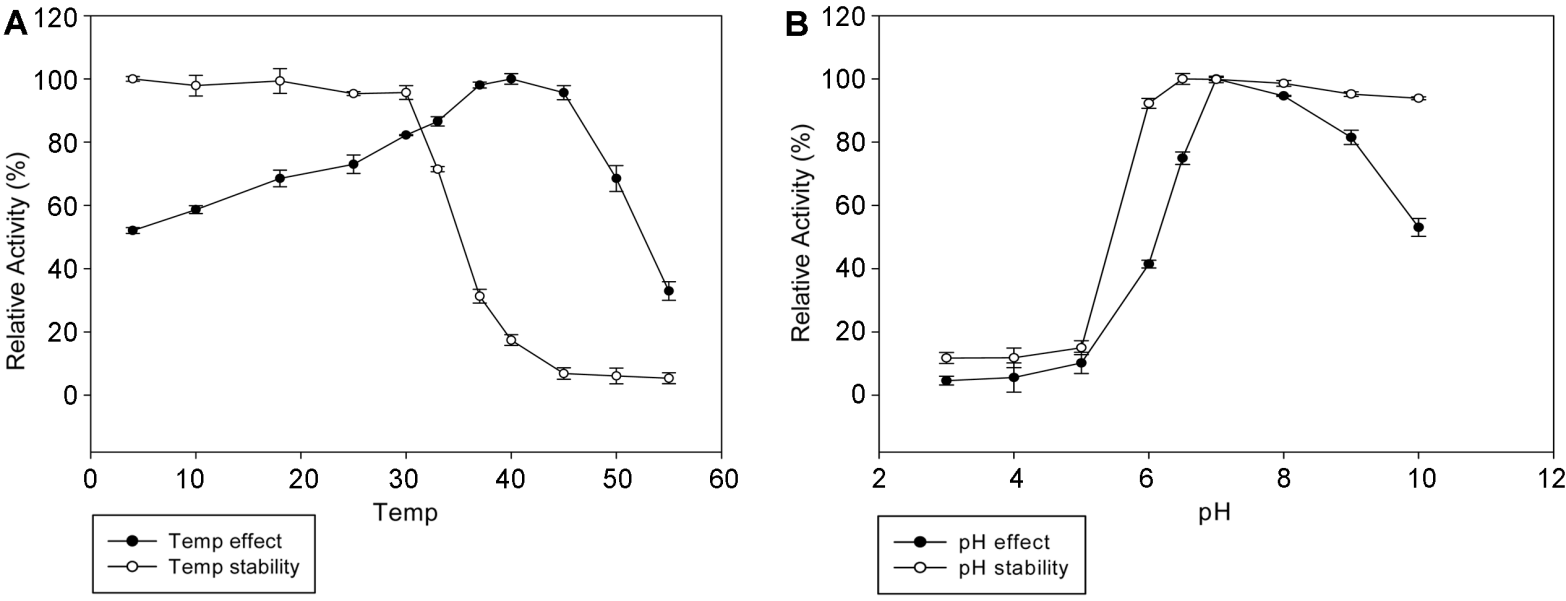
Effects of temperature (**A**) and pH (**B**) on the stability and activity of recombinant AbpBs.

The optimal temperature for AbpBs activity was 40°C and the enzyme was stable at lower than 30°C. The enzyme lost 65% of its activity at 55°C (Fig. 3A). The enzyme activity retained more than 75% of its optimal activity from pH 6.5 to 9.0, while pH 10.0 enzyme activity decreased by more than 40% and at under pH 5.0 the enzyme activity decreased to 20% (Fig. 3B). Additionally, the effects of metal ions, EDTA, β-mercaptoethanol of AbpBs activity were investigated (Table 1). AbpBs activity was weakly affected by β-mercaptoethanol in accordance with concentration, which is well known of thiol group inhibitors. These results suggested that sulfhydryl groups may be involved in the catalytic center of the enzyme [27-29]. The enzyme did not require Mg^2+^ for activity and was enhanced by 1 mM of Na^+^, K^+^, Mg^2+^, Mn^2+^, Ca^2+^, and Co^2+^. However, AbpBs activity was significantly inhibited by 10 mM EDTA, which indicated that divalent cations are required for enzymatic activity [30, 31]. The substrate specificity of AbpBs was tested using 1 mM *p*NP- and *o*NP-glycosides with α and β configurations described [25]. The results showed that AbpBs was only active against *p*NPAb; the other substrates, including *p*NP-β-D-glucopyranoside, *p*NP-N-acetyl-β-D-glucosaminide, *p*NP-β-D-mannopyranoside, *p*NP-β-D-xylopyranoside, *p*NP-α-D-glucopyranoside, *p*NP-α-L-rhamnopyranoside, *p*NP-α-D-mannopyranoside, *p*NP-α-D-xylopyranoside, *o*NP-β-D-glucopyranoside, *o*NP-β-D-galactopyranoside, and *o*NP-α-D-galactopyranoside, were not hydrolyzed. The K_m_ and V_max_ for the hydrolysis of *p*NPAb by AbpBs were 0.078 ± 0.0002 μM and 1.4 ± 0.1 μmol/min/mg/ of protein, respectively.

### Biotransformation of Ginsenoside Rb_2_

**Fig. 4:**
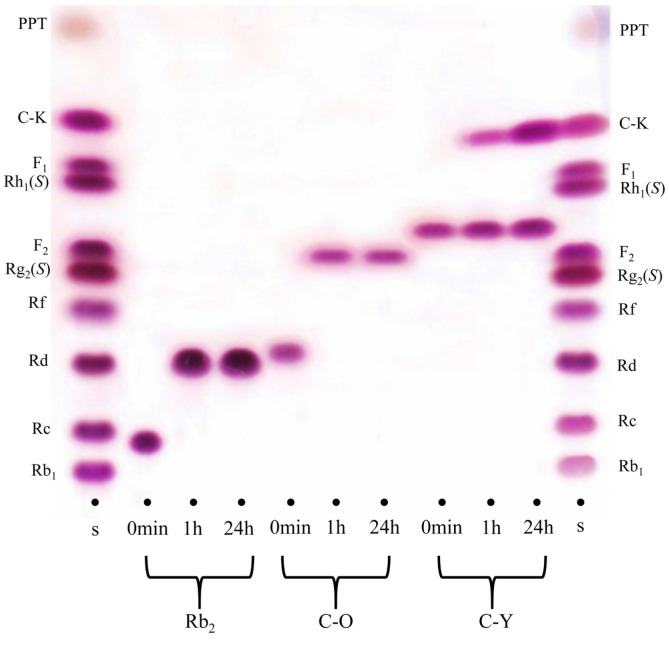
Time-course thin layer chromatography (TLC) analyses of biotransformation of Rb_2_, C-O, C-Y by recombinant AbpBs.

**Fig. 5:**
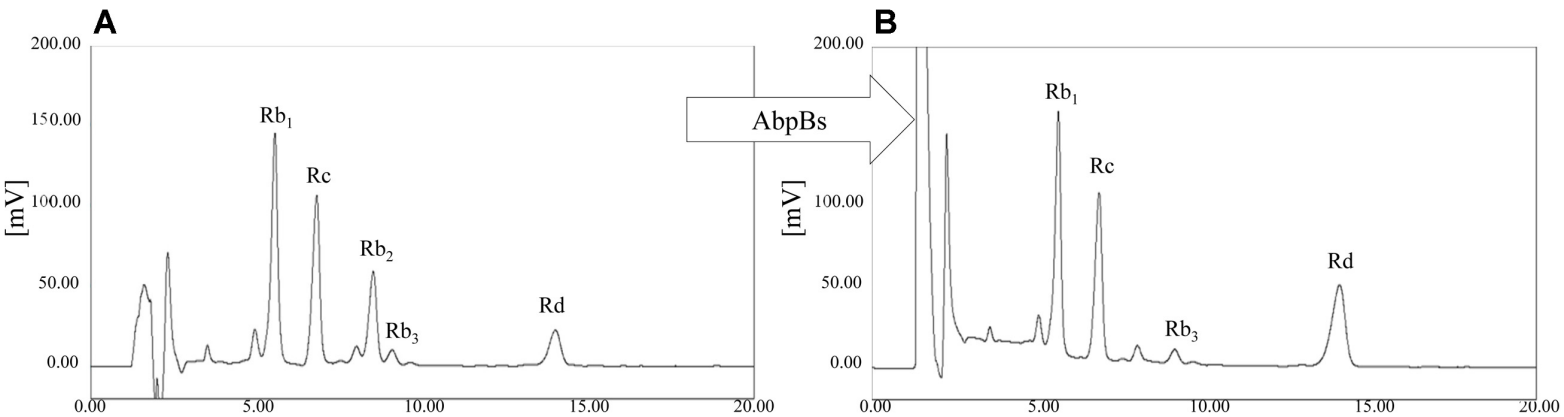
HPLC analysis results of transformation of PPD mixture by AbpBs. (**A**) Chromatogram of PPD mixture of *Panax ginseng*; (**B**) chromatogram of reaction mixture after 1 h.

For verification of the bioconversion of ginsenoside Rb_2_ into Rd by GST-AbpBs, TLC analyses were carried out at regular intervals (Fig. 4). It is clear that GST-AbpBs could completely transform the ginsenosides Rb_2_ and C-O into ginsenoside Rd and F_2_, respectively within 1 h. However, ginsenoside compound Y was partially converted to compound K regardless of long incubation time (Fig. 4). The HPLC chromatogram of a PPD mixture comprised of major ginsenosides (Rb_1_: 36.0%, Rc: 28.6%, Rb_2_: 17.0%, Rb_3_: 2.4%, Rd: 8.9%) was changed due to biotransformation following GST-AbpBs treatment. Ginsenoside Rb_2_ was completely converted to Rd within 1 h. These HPLC results indicate that AbpBs selectively converts ginsenoside Rb_2_ into Rd, but does not catalyze the hydrolysis of glucopyranosyl groups of Rb_1_ or other ginsenosides such as Rc, Rb_3_, and Rd (Fig. 5). The K_m_ and V_max_ for the hydrolysis of the α-L-arabinopyranose moiety by AbpBs were 0.92 ± 0.01 μM and 61.3 ± 1.5 μmol/min/mg/ of protein, respectively. These results show that AbpBs activity is faster for ginsenoside Rb_2_ than *p*NP- α-L-arabinopyranoside.

## Discussion

Until now, only one ginsenoside Rb_2_ hydrolyzing α-Larabinopyranosidase (Bgp2) belonging to the glycoside hydrolase family 2 had been reported [32]. However, this Bgp2 has a side effect of cleaving the glucose moiety of C20, such that it produces ginsenoside Rg_3_ and Rh_2_. Compared to Bgp2, AbpBs has high stereo activity, making it unable to hydrolyze glucose moiety of any ginsenoside. Ginsenoside Rb_2_ is one of the major PPD-type ginsenosides and accounts for 1–22% of the total ginsenosides in ginseng root or hair [18, 19], so a sizable amount can be potentially exploited. Ginsenoside Rd, produced by AbpBs, is an important divergent position during the biotransformation of ginsenosides, Rd → Rg_3_(*S*) → Rh_2_(*S*) pathway or Rd → F_2_ → compound K pathway (Fig. 1A), generated by the hydrolysis of the terminal or inner glucose moiety attached to the C3 or C20 carbon of ginsenoside Rd using β- glucosidase [33-36]. Thus, biotransformation of ginsenoside Rb_2_ to Rd by recombinant AbpBs would finally lead to the transformation of ginsenoside Rb_2_ to its most deglycosylated form, namely F_2_, Rg_3_(*S*), C-K or Rh_2_(*S*), if the appropriate β- glucosidase is used in combination with it.

In summary, a recombinant ginsenoside-hydrolyzing α- L-arabinopyranosidase (AbpBs) belonging to the glycoside hydrolase family 2 was cloned from *Blastococcus saxobsidens* and constructed for biotransformation of the major ginsenoside Rb_2_. The enzyme acts optimally at pH 7.0 and 40°C. AbpBs could hydrolyze Rb_2_, C-O, C-Y into Rd, F_2_, CK, respectively, by selectively hydrolyzing the outer arabinopyranoside moiety at the C20 position. Biotransformed ginsenoside Rd and other minor ginsenosides derived from it are potentially useful in the cosmetic and pharmaceutical industry.

## Figures and Tables

**Table 1:** Effects of metal ions and other chemical agents on the activity of purified AbpBs.

Metal ions or reagents	Relative activity ± SD (%)	

	1 mM	10 mM
NaCl	111.1 ± 1.4	124.6 ± 1.1
KCl	125.5 ± 3.1	183.4 ± 1.0
MgCl_2_	114.5 ± 2.5	97.5 ± 1.4
CoCl_2_	109.3 ± 1.7	111.4 ± 1.6
CaCl_2_	116.6 ± 2.6	113.3 ± 1.7
MnSO_4_	111.5 ± 3.3	112.2 ± 1.5
MgSO_4_	112.6 ± 2.9	83.7 ± 0.9
EDTA	80.2 ± 1.2	37.6 ± 2.5
β-Mercaptoethanol	111.1 ± 1.4	85.0 ± 0.7
None	100 ± 2.2	100 ± 1.3
